# Neurodevelopmental Outcomes and Gut Bifidobacteria in Term Infants Fed an Infant Formula Containing High sn-2 Palmitate: A Cluster Randomized Clinical Trial

**DOI:** 10.3390/nu13020693

**Published:** 2021-02-22

**Authors:** Wei Wu, Ai Zhao, Biao Liu, Wen-Hui Ye, Hong-Wen Su, Jing Li, Yu-Mei Zhang

**Affiliations:** 1 Department of Nutrition and Food Hygiene, School of Public Health, Peking University, Beijing 100191, China; wennie0616@126.com; 2 Venke School of Public Health, Tsinghua University, Beijing 100091, China; xiaochaai@163.com; 3 Milk Powder BU, Inner Mongolia Yili Industrial Group Co., Ltd., Hohhot 010000, China; bliu@yili.com (B.L.); nfywh@yili.com (W.-H.Y.); nfsuhongwen@yili.com (H.-W.S.); lijing02@yili.com (J.L.)

**Keywords:** sn-2 palmitate, infant, Bifidobacteria, neurodevelopment, fine motor

## Abstract

A few studies suggested high stereo-specifically numbered (sn)-2 palmitate in a formula might favor the gut Bifidobacteria of infants. The initial colonization and subsequent development of gut microbiota in early life might be associated with development and later life functions of the central nervous system via the microbiota–gut–brain axis, such as children with autism. This study aims to assess the hypothesized effect of increasing the amount of palmitic acid esterified in the sn-2 position in infant formula on neurodevelopment in healthy full-term infants and to explore the association of this effect with the altered gut Bifidobacteria. One hundred and ninety-nine infants were enrolled in this cluster randomized clinical trial: 66 breast-fed (BF group) and 133 formula-fed infants who were clustered and randomly assigned to receive formula containing high sn-2 palmitate (sn-2 group, *n* = 66) or low sn-2 palmitate (control group, *n* = 67), where 46.3% and 10.3% of the palmitic acid (PA) was sn-2-palmitate, respectively. Infants’ neurodevelopmental outcomes were measured by the Ages and Stages Questionnaire, third edition (ASQ-3). Stool samples were collected for the analysis of Bifidobacteria (Trial registration number: ChiCTR1800014479). At week 16, the risk of scoring close to the threshold for fine motor skills (reference: scoring above the typical development threshold) was significantly lower in the sn-2 group than the control group after adjustment for the maternal education level (*p* = 0.036) but did not differ significantly versus the BF group (*p* = 0.513). At week 16 and week 24, the sn-2 group (week 16: 15.7% and week 24: 15.6%) had a significantly higher relative abundance of fecal Bifidobacteria than the control group (week 16: 6.6%, *p* = 0.001 and week 24:11.2%, *p* = 0.028) and did not differ from the BF group (week 16: 14.4%, *p* = 0.674 and week 24: 14.9%, *p* = 0.749). At week 16, a higher relative abundance of Bifidobacteria was associated with the decreased odds of only one domain scoring close to the threshold in the formula-fed infants group (odds ratio (OR), 95% confidence interval (CI): 0.947 (0.901–0.996)). Elevating the sn-2 palmitate level in the formula improved infants’ development of fine motor skills, and the beneficial effects of high sn-2 palmitate on infant neurodevelopment was associated with the increased gut Bifidobacteria level.

## 1. Introduction

Human milk (HM) is the optimal food for healthy infants and provides adequate nutrients to support growth. However, some infants need formula when breastfeeding is not available. Triacylglycerol (TAG), the major source of energy in both HM and infant formula (IF), is comprised of three fatty acids (FAs) selectively esterified to the glycerol skeleton at three stereo-specifically numbered (sn) positions [1]. Different TAG structures in IF, compared to HM, have been found to contribute to the differences in gastrointestinal (GI) symptoms related to feeding patterns. Palmitic acid (PA), an abundant saturated FA in both HM and IF (approximately 20% of the total FAs), is predominantly esterified (approximately 70%) at the sn-2 (center carbon) position in HM but primarily esterified at the sn-1 and sn-3 (outer carbon) positions in fat blends commonly used in IF [2]. During TAG digestion, the pancreatic lipase–colipase system preferentially hydrolyzes FAs in the sn-1 and sn-3 positions, yielding two free FAs and the corresponding sn-2 monoglyceride [3]. All sn-2 monoglycerides are well-absorbed [4]; however, saturated free FAs with chain lengths >12 carbons, such as PA, are easily present in the intestinal lumen and bind calcium to form FA soaps. These FA soaps are insoluble and indigestible in the intestine and, thus, end up in the colon to be excreted along with the feces, which are associated with hard, infrequent stools [5]. Therefore, PA in HM is well-absorbed, given its predominant esterification in the sn-2 position. The IF rich in sn-2 palmitate yields a positional distribution of PA that more closely resembles that of HM. With less FA soaps present in feces, HM-fed infants and infants who consumed a formula containing a high level of sn-2 palmitate had softer stools, which were easy to pass, and less gastrointestinal (GI) symptoms [6,7,8], thus promoting GI health. Other effects of high sn-2 palmitate on infant health remain to be corroborated.

Regarding the benefits on GI health, high sn-2 palmitate in a formula may also have potential prebiotic effects. Bacterial genera frequently exploited as probiotics include Bifidobacterium and Lactobacillus. Yaron et al. found that term infants fed formula containing high sn-2 palmitate had higher Bifidobacteria counts than those fed formula containing low sn-2 palmitate after six postnatal weeks [7]. In another study on healthy term infants, a significantly higher fecal Bifidobacteria concentration was also observed in the high sn-2 palmitate group compared to the control at week eight, not differing from HM-fed infants [9]. However, whether the possible effect of high sn-2 palmitate on promoting the gut Bifidobacterium of infants persists longer remains unknown.

Positive colonization featuring a higher abundance of Bifidobacteria at birth confers favorable effects throughout the whole life [10,11,12]. Notably, the role of the initial colonization and subsequent development of gut microbiota in early life plays a role in development, and later life functions of the central nervous system (CNS) have been revealed in a growing number of studies [13,14,15,16], and the effect pathway is recognized as the microbiota–gut–brain axis (MGBA) [17]. In human populations, a few studies have suggested that probiotics can relieve symptoms of certain neuropsychiatric diseases by altering the gut microbiota composition [18,19]. However, it is still unexplored whether the potential prebiotic effect of high sn-2 palmitate is associated with the neurodevelopment in early life via the MGBA.

To assess the hypothesized effect of increasing the amount of PA esterified in the sn-2 position in IF on neurodevelopment in early life, and to explore the association of this effect with an alteration in the gut Bifidobacteria, we evaluated the fecal Bifidobacteria and neurodevelopment in healthy full-term infants receiving a control formula versus a formula with an elevated level of sn-2 palmitate.

## 2. Materials and Methods

### 2.1. Study Design

This was a cluster-randomized, controlled, parallel-group study conducted in China of healthy, term formula-fed infants receiving one of two study formulas for 24 weeks. A nonrandomized breast-fed (BF) group was included as a reference. Clinic visits were scheduled at baseline, week 16, and week 24, with telephone follow-ups midway between clinic visits.

All the legal guardians of each infant gave their informed consent for inclusion before the infant participated in the study. The study was conducted in accordance with the Declaration of Helsinki, and the protocol was approved by the Medical Ethics Research Board of Peking University (No. IRB00001052-17081, 20 December 2017). This study has been registered in the Chinese Clinical Trial Registry (Registration number: ChiCTR1800014479).

### 2.2. Participants

Infants were enrolled by medical staff from four obstetric units between December 2017 and January 2019. Infants were healthy, term (gestational age ≥37 weeks), singleton, aged 7 to 14 days, birth weights between 2500 and 4500 g, and exclusively consuming and tolerating a cow’s milk–based IF (for the formula-fed groups) or exclusively breastfeeding (for the BF group). Only when the mother had unequivocally decided not to breastfeed was she approached for inclusion of her infant in the formula-fed groups. The main exclusion criteria were infants with genetic or congenital diseases, receiving antibiotic therapy, requiring hospitalization for longer than 7 days, having familial history for atopy, and metabolic or chronic diseases. Infants who additionally received antibiotics, prebiotics, or probiotics (except the component of galacto-oligosaccharide in the study formulas) at any time throughout the study period were not included in the data analyses.

### 2.3. Study Feedings

Infants enrolled in the BF group continued to receive HM ad libitum. Formula-fed infants from the four obstetric units were assigned to receive one of the following study formulas ad libitum according to a cluster randomization process based on the four blocks, created by an external statistician using sealed envelopes. After randomization, formula-fed infants were to consume the assigned formula exclusively until the introduction of complementary feeding. The composition of the two formulas was comparable, with the exception of the sn-2 palmitate level: sn-2, a high sn-2 palmitate IF in which 46.3% of the PA was esterified to the sn-2 position and control, an IF containing a standard vegetable oil mixture, in which 10.3% of the PA was esterified to the sn-2 position. The content of the sn-2 PA level in the high sn-2 palmitate IF was increased by adding refined vegetable fat, Betapol B-55 DMX (1,3-Dioleoyl-2-Palmitoy-TAG), produced by Bunge Loders Croklaan Oils (Pasir Gudang, Malaysia). Both study formulas followed the National Food Safety Standard on Infant Formula and the nutritional recommendations for infants [20]. FA profiles, the percentage of sn-2 palmitate, and selected nutrient levels in the formulas are listed in Table 1. Formula labels provided preparation and storage instructions in Chinese.

### 2.4. Data Collection

#### 2.4.1. General Information

Demographic and household characteristics were collected at the baseline clinic visit. A parent questionnaire about information of the infant’s health condition (illness, medication, and hospitalization) and GI symptoms was filled out at the baseline, week 16, and week 24 visits.

A food frequency questionnaire was used to investigate the feeding pattern and the complementary food consumed by infants. Parents were also given a diary to record the volume of study formula consumed for the 2 days immediately prior to each of the clinic visits.

#### 2.4.2. Ages and Stages Questionnaire

Infants’ developmental outcomes were measured by the Ages and Stages Questionnaire, third edition (ASQ-3) [21]. With the aid of medical staff, the ASQ-3 was administered by parents during the week 16 and week 24 visits. The ASQ-3 is a validated monitoring instrument used to screen infants and toddlers aged between 1 month and 66 months for the risk of a developmental delay across 5 developmental domains: gross motor skills, fine motor skills, problem-solving ability, communication, and personal and social skills. Each domain is assessed by 6 questions ascertaining the achievement of relevant skills. Scores for individual items are summed to give an overall continuous score for each of the 5 domains (a possible range of 0–60). Compared with the normal reference of Chinese infants [22], the ASQ-3 scores were also categorized according to a priori recommendations of 3 categories: scoring above, close to (between the mean and mean-2 standard deviation (SD)) or below (below the Mean-2SD) the typical development threshold of similar-aged infants [21], indicating typical development, a need for monitoring, or a need for further assessment, respectively. We also categorized the number of domains scoring close to and below the threshold into 3 categories: 0, 1, and ≥2.

#### 2.4.3. Anthropometry

Infant weight and length were obtained at the baseline, week 16, and week 24 visits. Infants were weighed without clothing or diaper on an electronic infant scale, and measurements were recorded to the nearest 10 g. Recumbent length was measured on a pediatric length board to the nearest 0.1 cm. Measures were taken twice at each visit, and the mean was calculated.

#### 2.4.4. Fecal Bifidobacteria

A fresh stool sample (~1 g) was collected from infants by parents/caregivers at home during a 2-day period before the baseline, week 16, and week 24 visits. Samples were collected with a scoop attached to the inner aspect of the lid of a sterile polypropylene vial and immediately stored in a −20 °C freezer. Frozen samples were shipped by cold chain prior to being analyzed with 16S high-throughput sequencing for microbial genomes. DNA was isolated from the fecal samples by using a Qiagen QIAamp DNA Stool Mini Kit (Qiagen, Duesseldorf, Germany) and were stored at −20 °C. The V4 regions of the bacteria 16S rRNA gene were amplified by using primers 515F (5′-barcode-GTGCCAGCMGCCGCGGTAA-3′) and 806R (5′-barcode-GGACTACHVGGGTWTCTAAT-3′) with 95 °C lasting 2 min; 25 cycles of 95 °C for 1 min, 55 °C for 1 min, and 72 °C for 1 min; and 72 °C for a final 5 min. Amplicons were purified and quantified by using the Qiagen QIAquick PCR purification kit (Qiagen, Duesseldorf, Germany) and PicoGreen^®^ double-stranded DNA (dsDNA) reagent kit (Invitrogen Ltd., Paisley, UK), respectively. Purified amplicons were pooled and were sequenced on an Illumina MiSeq platform. Then, the paired-end reads were merged using pandaseq and then assigned to each sample based on their unique barcodes. High-quality clean tags were clustered into operational classification units (OTUs) using USEARCH based on a 97% sequence similarity using QIIME. Representative OTUs were used for further analysis using the SILVA bacterial database with the RDP algorithm. Data were reported as the relative abundance of Bifidobacteria.

#### 2.4.5. Digestive Tolerance

Infants’ digestive tolerance and behaviors were recorded during clinic visits and telephone calls throughout the study. A subset of symptoms relating to the digestive system were identified of particular interest a priori: difficulty having a bowel movement, regurgitation, vomiting, bowel cramps, abdominal distension, acute diarrhea, and chronic diarrhea. The severity of symptoms, and whether they were related to the study feedings, were determined by the investigators. To ensure consistency in diagnosis, investigators were provided with standard definitions of these symptoms.

### 2.5. Statistical Analysis

Data analysis was conducted as outlined in an a priori statistical analysis plan using SAS software version 9.4 (SAS Institute, Cary, NC, USA). Data were described as the mean ± standard deviation or median (25th and 75th percentiles) for continuous variables and by frequency (percentage %) for categorical variables.

Baseline characteristics were compared between two groups by the paired samples test, Wilcoxon Rank test, or chi-square test. The prespecified comparison of primary interest was between the sn-2 group and the control group. Subsequently, these two formula groups were compared with the BF group, respectively. The primary outcome was fecal Bifidobacteria. Between-group differences with a relative abundance of fecal Bifidobacteria were analyzed using the Wilcoxon Rank test. Differences in ASQ-3 scores between the two groups were analyzed using a partial correlation analysis, with the maternal education level included as the covariate. Multinomial logistic regression was conducted to investigate the risk of scoring close to and below the typical development threshold for five domains, adjusted for the maternal education level, and the analyses were repeated, with the number of domains scoring close to and below the threshold. Potential correlations of these findings were explored by a multinomial logistic regression, with treatment and maternal education level included in the model. A *p*-value <0.05 was considered to be statistically significant.

Compliance was evaluated by comparing the quantity of formula consumed between the two formula-fed groups and the complementary food consumed among three groups. All analyses were performed applying the per-protocol (PP) principle.

## 3. Results

### 3.1. Study Population

**Figure 1:**
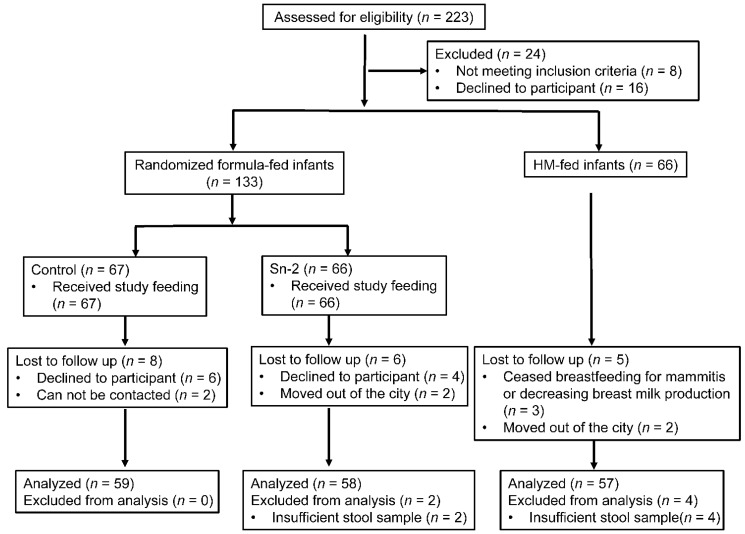
CONSORT flowchart from enrolment to analysis. sn-2 = the high sn-2 palmitate infant formula, in which 46.3% of the palmitic acid (PA) was esterified to the sn-2 position, BF = breast-fed, and Control = the infant formula, in which 10.3% of the PA was esterified to the sn-2 position.

A total of 133 formula-fed infants were randomly assigned to receive control or sn-2 formulas (Figure 1). Sixty-six BF infants were enrolled. Of the infants enrolled, 174 completed the study. The demographic and clinical characteristics were mostly comparable among the three groups (Table 2). No significant differences in the maternal or infant characteristics were observed between the formula-fed groups. The percentage of infants of vaginal delivery and maternal education level were significantly higher in the BF group than both the sn-2 group and the control group. Although more mothers in the BF group had a normal body mass index (BMI) (18–24) before pregnancy than both of the two formula-fed groups, the differences were not statistically significant.

Compliance was similar and continuous for the two formula-fed groups as infants in both groups were reported to exclusively formula feed at week 16 (125.7 g/day vs. 129.2 g/day) and week 24 (158.0 g/day vs. 165.3 g/day).There were three (5.2%), four (6.8%), and four (7.0%) infants reported to consume complementary food at week 24 in the sn-2 group, the control group, and the BF group, respectively. Among these infants, the average time when complementary food was first introduced was at week 23.3, week 22.3, and week 22. The complementary foods were cereals (including rice flour and noodles) and fresh fruits. There were no significant differences in the percentage of infants, the first time, and the kinds of complementary foods among the three groups.

### 3.2. Anthropometric Data

No significant differences were observed in the infants’ weights, lengths, or head circumferences at the baseline or during any visit (Supplementary Table S1).

### 3.3. ASQ-3 Scores

At week 16, a comparison of the ASQ-3 scores showed the median score for fine motor skills in the sn-2 group (50.0 (45.0, 60.0)) was significantly higher than the control group (45.0 (35.0, 55.0), *p* = 0.021) after adjustment for the maternal education level but did not differ significantly versus the BF group (55.0 (45.0, 60.0), *p* = 0.096) (Supplementary Table S2). Similarly, with scoring above the typical development threshold as a reference, the risk of scoring close to the threshold for fine motor skills was significantly lower in the sn-2 group than the control group, and this remained significant even after the adjustment for the maternal educational level (Table 3). Although we observed a higher risk of scoring close to the threshold for personal and social skills in the sn-2 group compared to the BF group, this was not statistically significant when adjusted for the maternal educational level (Table 3).The differences between the control group and the BF group on the scores of fine motor, problem-solving, and the risk of only one domain scoring close to the threshold were also attenuated by adjustment for the maternal educational level.

At week 24, there were no significant differences in any domains of the ASQ-3 among the three groups (Supplementary Tables S3 and S4).

### 3.4. Fecal Bifidobacteria

**Figure 2:**
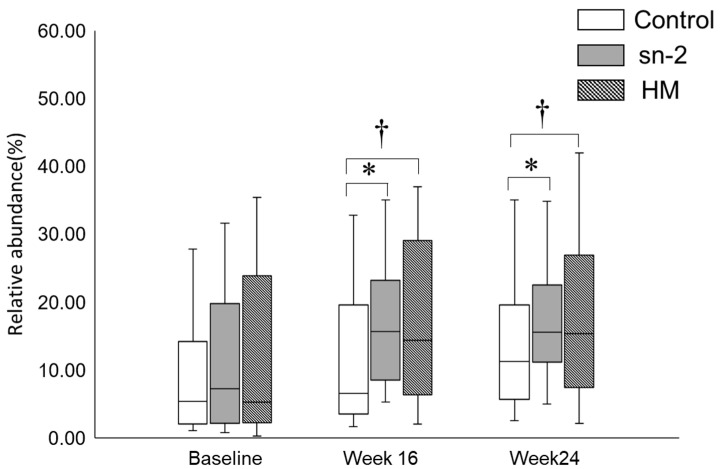
Relative abundance of Bifidobacteria at baseline, week 16, and week 24 among the feeding groups. (1) Values are median (25th and 75th percentiles). (2) * The difference was significant between the sn-2 group and the control group at week 16 (*p* = 0.001) and week 24 (*p* = 0.028) by the Wilcoxon Rank test. ^†^ The difference was significant between the control group and the BF group at week 16 (*p* = 0.029) and week 24 (*p* = 0.020) by the Wilcoxon Rank test. sn-2 = the high sn-2 palmitate infant formula, in which 46.3% of the PA was esterified to the sn-2 position, BF = breast-fed, and Control = the infant formula, in which 10.3% of the PA was esterified to the sn-2 position.

Figure 2 shows the relative abundance of fecal Bifidobacteria at the baseline, week 16, and week 24. At the baseline, the Bifidobacteria relative abundance did not differ significantly among the sn-2 group, the control group, and the BF group. After 16 weeks and 24 weeks, infants fed the control formula had significantly lower Bifidobacteria relative abundance compared to those fed the high sn-2 palmitate formula or breast-fed. The Bifidobacteria relative abundance in the sn-2 group did not differ from that in the BF group at week 16 or week 24.

In both the subgroups stratified by the delivery mode, comparing the Bifidobacteria relative abundance among the three groups showed the same trend with the analysis of all infants at the baseline, week 16, and week 24.

To explore the possible associations among these findings, we assessed the alteration in the odds of scoring close to or below the typical development threshold for five domains of the ASQ-3 per one percentage increase in the relative abundance of the fecal Bifidobacteria in formula-fed infants at week 16 (Table 4). No significant associations with the Bifidobacteria relative abundance were found in each domain. We performed a further analysis on the number of domains scoring close to and below the threshold. With “zero” as the reference, the decreased odds of only one domain scoring close to the threshold was associated with a higher relative abundance of Bifidobacteria.

### 3.5. Digestive Tolerance and Allergic Diseases

No significant differences in GI symptoms related to digestive tolerance were observed among the sn-2 group, the control group, and the BF group at week 16: difficulty having a bowel movement (zero (0%), zero (0%), and one (1.7%)); abdominal distension (zero (0%), two (3.4%), and one (1.8%)); and acute diarrhea (four (6.9%), two (3.4%), and zero (0%)). The same situation was observed at week 24. Bowel cramps were slightly more common in formula-fed infants than BF infants at both week 16 (6 (10.7%), 12 (20.3%), and 1 (1.8%)) and week 24 (five (8.6%), four (6.8%), and zero (0%)), while none of these differences met the significant threshold. In infants who experienced these GI symptoms, 93.3% were unrelated to feedings and were mild in intensity.

Allergic diseases were reported in four (6.9%), five (8.5%), and two (3.5%) infants in the sn-2 group, the control group, and the BF group, respectively, at week 16, as well as in four (6.9%), one (1.7%), and seven (12.7%) at week 24.

## 4. Discussion

Clinical studies have demonstrated the effects of increasing the sn-2 palmitate content of IF on improving the FA and calcium absorption, bone health, gut Bifidobacteria abundance, and infant GI comfort [4,6,7,8,9]. However, the effects on neurodevelopment have received little attention. In this study, the experimental formula contained 46.3% of PA as sn-2 palmitate, an intermediate level between HM and the control formula (10.3%). To the best of our knowledge, this is the first study that found out infants fed a formula with high sn-2 palmitate for 16 weeks had higher scores and a lower risk of scoring close to the typical development threshold for fine motor skills than the control formula. Our findings also suggested that the favorable effect of high sn-2 palmitate on infant neurodevelopment was associated with the increased gut Bifidobacteria relative abundance.

In terms of neurodevelopment, even modest decrements in the average ASQ-3 performance have potential clinical and public health importance, as a modest decrement in the mean ASQ-3 score across children with predominantly typical neurodevelopment could disproportionately affect the prevalence of abnormal scores, which is correlated with developmental disturbances [23]. In this study, our analyses revealed a lower risk of a possible developmental delay in fine motor skills in infants fed the high sn-2 palmitate formula for 16 weeks than the control formula in the unadjusted model and a same result in the model adjusted for the maternal education level, although the maternal education level did not differ significantly between the two formula-fed groups. However, the maternal education level was higher in the nonrandomized BF group than both the formula-fed groups. The association of exclusively breastfeeding under six months with the maternal education level has been found in previous studies [24,25]. A higher risk of scoring close to the threshold for personal and social skills in the sn-2 group than the BF group was observed without adjustment for the between-groups differences in the maternal education level, which was also reported to be positively associated with the ASQ-3 score in another study [26]. Fine motor skills may serve as an indicator for cognitive skills, such as visual information processing [27]. We did not observe a difference in the performance of fine motor skills at week 24. The reason might be that, after being informed of the assessment result of the ASQ-3 at the week 16 visit, some caregivers might have conducted targeted training on the domain with the risk of development delay between these two visits. This might, to some extent, attenuate the difference in the fine motor skills development between the two groups at the 24-week visit.

The sn-2 palmitate enrichment in formula might have direct effects on infant neurodevelopment due to the higher and more efficient PA absorption, which might cause polyunsaturated fatty acids to be used less as fuel and, thus, to be more available for membrane incorporation of the CNS [28]. A further analysis in our study suggested the effect of high sn-2 palmitate in formula on infant neurodevelopment might be correlated with its prebiotic effect. The relative abundance of fecal Bifidobacteria was found to be associated with the odds of only one ASQ-3 domain scoring close to the typical development threshold among formula-fed infants at week 16, although no direct associations with the fine motor domain were observed. Some previous studies only observed associations of the gut microbiome composition with neurodevelopment outcomes in older children and did not focus on probiotics. Sordillo observed an association between the gut microbiome composition from infants aged three to six months and their fine motor skills, evaluated by the ASQ-3 at three years of age, in a cohort study [26]. In another prospective study among 69 infants, the cognitive outcomes on language at two years of age differed between the three clusters of microbial compositions at one year of age [29]. Our study suggested that the association of infant gut microbes with neurodevelopment can be captured as early as infancy. Coinciding with this critical window of gut microbiota shaping, the first years postnatally also represent a crucial time of early brain development [30], when several foundational processes are simultaneously occurring in the brain, including proliferation, apoptosis of the glia, myelination of the axons, and synaptogenesis [31]. These processes are accompanied by the rapid emergence of various cognitive functions [32]. It is increasingly recognized that the gut microbiota regulates infantile neurodevelopment and recognition function through MGBA. This gut–brain axis represents a complex network of communication between the GI tract and the CNS [33]. It encompasses the CNS and the sympathetic and parasympathetic branches of the autonomic nervous system, as well as the enteric nervous system and the neuroendocrine and neuroimmune systems. A number of mechanisms have been postulated to underlie how MGBA functions, including the microbiome’s role in immune system development and regulation [34]. In addition, signals originating from the gut microbiota to the brain are likely delivered by bacterial metabolites, such as neuroactive short-chain FAs [35,36]. Bacteria also produce neurotransmitters that may be involved with central neural pathways by interfering with host transmitter functions [37]. Therefore, it is reasonable to speculate that the gut Bifidobacteria might function under the beneficial effect of high sn-2 palmitate on infant neurodevelopment observed in our study by the MGBA.

Early life perturbations of the developing gut microbiota can negatively influence neurodevelopment. Dysbiosis of the gut microbiota can affect the exchange of information within the MGBA and is linked to neuropsychiatric disorders in children, such as autism spectrum disorder (ASD) and multiple sclerosis [15,16]. A handful of population studies demonstrated that probiotics can relieve certain neuropsychiatric diseases by altering the gut microbiota composition [18,19], indicating that different bacterial genera species may play different roles in regulating the neurodevelopment and function [38]. Shaaban observed increases in the colony counts of Bifidobacteria and lactobacilli levels in children with ASD, with significant improvements in the severity of autism, after three months of supplementation of a probiotic nutritional supplement formula [39]. As probiotics were reported to be useful in the treatment of certain neuropsychiatric diseases, probiotics such as Bifidobacteria may well benefit neurodevelopment in early life.

Our finding that infants fed the high sn-2 palmitate formula had a higher relative abundance of fecal Bifidobacteria than the control formula supported the possible mediation of Bifidobacteria during the effect of high sn-2 palmitate on infant neurodevelopment. An increased Bifidobacteria relative abundance in the sn-2 group is also consistent with previous studies demonstrating that high sn-2 palmitate in IF favored the Bifidobacteria colonies after infants were fed for six or eight weeks [7,9]. Our study further observed that this effect can persist for 24 weeks. The mechanism of the prebiotic effect of high sn-2 palmitate in formula is still unclear. The support of beneficial bacterial populations may result from indirect growth inhibition of these populations by palmitate–calcium soaps or fat degradation products that activate certain metabolic pathways [7]. Further investigation is needed in this area.

Nevertheless, despite the interesting results, we are aware of the study limitations. Firstly, we did not collect and analyze the HM samples from the BF group but referred to the sn-2 palmitate level in the HM of previous studies. Secondly, based on the study design of randomization, we investigated a number of potentially important variables, including dietary factors and the demographic and clinical characteristics of subjects. We also adjusted the maternal education level and the treatment in the model; however, other environmental factors that might influence the neurodevelopment were not measured in the current study but would represent a valuable addition to future studies. Thirdly, considering the withdrawals all occurred within eight weeks after the baseline visit, we adopted the PP analysis instead of the intention-to-treat analysis, which might somewhat have influenced the observed effects. The study design could also limit the generalizability of our results to some extent, as the association of high sn-2 palmitate in a formula with infant neurodevelopment was captured at week 16 after birth and in a population of which the majority had typical neurodevelopment. The long-term significance of this association warrants further confirmation in prospective studies of large populations.

## 5. Conclusions

This study provided new insights into the benefits of increasing the level of sn-2 palmitate in IF on infants’ health outcomes. Elevating the sn-2 palmitate level in the formula improved infants’ development of fine motor skills, and the beneficial effects of high sn-2 palmitate on infant neurodevelopment was associated with the increased gut Bifidobacteria level. These findings further emphasized the importance of fat structures in HM and IF and may have inspiring implications regarding the potential for dietary interventions to attenuate the neurodevelopmental risk.

## Figures and Tables

**Table 1:** Compositions of the study formulas.

Composition	sn-2	Control
Energy, KJ/100 g	2130.3	2102.0
Protein, g/100 g	11.7	10.4
Fat, g/100 g	26.8	27.2
Carbohydrate, g/100 g	53.7	57.8
Magnesium, mg/100 g	30.0	38.0
Calcium, mg/100 g	349.4	380.0
Phosphorus, mg/100 g	223.7	230.0
Vitamin B6, μg/100 g	420.0	439.0
Vitamin B12, μg/100 g	1.5	1.5
Folic acid, μg/100 g	65.0	81.5
Pantothenic acid, μg/100 g	2850.0	2871.0
Biotin, μg/100 g	16.0	15.1
Galacto-oligosaccharide, g/100 g	1.5	1.5
Fatty acid (%) ^1^		
C10	0.5	0.8
C12	0.7	0.7
C14	2.1	2.5
C15	0.2	0.1
C16:0	25.8	24.6
C16:0 in sn-2 position ^2^	46.3	10.3
C16:1	0.2	0.1
C18	4.4	4.4
C18:1n9	35.2	37.1
C18:2n6	20.6	19.5
C18:3n6	0.1	0.1
C18:3n3	2.6	1.9
C20	0.3	0.4
C20:4n6	0.4	0.2
C22	0.2	0.3
C24	0.2	0.1

^1^ wt % of total fatty acids. ^2^ wt % of total C16:0. sn-2 = the high sn-2 palmitate infant formula by adding refined vegetable fat, Betapol B-55 DMX (1,3-Dioleoyl-2-Palmitoy-TAG), and Control = the infant formula containing a standard vegetable oil mixture.

**Table 2:** Baseline demographic and clinical characteristics by feeding groups ^1^.

Characteristic		Feeding Group		
		Control	sn-2	BF
***n***		59	58	57
			Infant	
Female (%)		30 (50.9)	25 (43.1)	21 (36.8)
Age, days		11 ± 1.7	12 ± 1.2	9 ± 2.5
Gestational age, weeks		38.6 ± 1.3	38.8 ± 1.0	39.2 ± 0.9
Vaginal delivery (%) ^2^		15 (25.4)	23 (37.5)	38 (66.7)
Weight, kg		3.4 (3.1, 3.6)	3.3 (3.1, 3.6)	3.4 (3.1, 3.6)
Length, cm		50.0 (50.0, 51.0)	50.0 (50.0, 51.0)	50.0 (50.0, 51.0)
			Mother	
Age, years		29 (27, 32)	31 (28, 35)	30 (28, 31)
Education level (%) ^3^ bachelor’s degree or above		12 (20.3)	15 (25.9)	31 (54.4)
Occupational status (%) working		34 (57.6)	31 (53.4)	40 (70.2)
BMI before pregnancy	≤18.0	11 (18.6)	7 (12.1)	7 (12.3)
	≤24.0	35 (59.3)	37 (63.8)	45 (78.9)
	>24.0	13 (22.0)	14 (24.1)	5 (8.8)
Smoking during pregnancy		1 (1.7)	1 (1.7)	1 (1.8)
Drinking during pregnancy		0 (0.0)	0 (0.0)	(0.0)

^1^ Data were described by the mean ± standard deviation or median (25th and 75th percentiles) for continuous variables and frequency (percentages %) for categorical variables. sn-2 = the high sn-2 palmitate infant formula, in which 46.3% of the PA was esterified to the sn-2 position, BF = breast-fed, and Control = the infant formula in which 10.3% of the PA was esterified to the sn-2 position. ^2^ A significant difference in the delivery mode was observed between the sn-2 group versus the BF group (*p* < 0.001) and the control group versus the BF group (*p* < 0.001) based on the chi-square test. ^3^ A significant difference in the maternal education level was observed between the sn-2 group versus the BF group (*p* = 0.003) and the control group versus the BF group (*p* = 0.002) based on the chi-square test. BMI = body mass index.

**Table 3:** Comparison of the Ages and Stages Questionnaire, third edition (ASQ-3) scores with the typical development threshold at 16 weeks among the feeding groups.

Developmental Domain	Compared with the Typical Development Threshold ^1^	Control 59	sn-2 58	BF 57	*p*					
					sn-2 vs. Control		sn-2 vs. BF		Control vs. BF	
					Unadjusted ^2^	Adjusted ^3^	Unadjusted ^2^	Adjusted ^3^	Unadjusted ^2^	Adjusted ^3^
Communication	Above	50 (84.8)	49 (84.5)	52 (91.2)	Reference		Reference		Reference	
	Close to	5 (8.5)	7 (12.1)	4 (7.0)	0.543	0.574	0.331	0.156	0.708	0.307
	Below	4 (6.8)	2 (3.4)	1 (1.8)	0.463	0.474	0.533	0.258		0.161
Gross motor	Above	53 (89.8)	56 (96.6)	56 (98.3)	Reference		Reference		Reference	
	Close to	3 (5.1)	2 (3.4)	1 (1.8)	0.635	0.687	0.566	0.304	0.324	0.127
	Below	3 (5.1)	0 (0.0)	0 (0.0)	0.954	0.950	—	—	0.954	0.950
Fine motor	Above	46 (78.0)	53 (91.4)	50 (87.7)	Reference		Reference		Reference	
	Close to	11 (18.6)	3 (5.2)	7 (12.3)	0.037	0.036	0.217	0.513	0.308	0.283
	Below	2 (3.4)	2 (3.4)	0 (0)	0.904	0.882	0.984	0.953	0.943	0.943
Problem-solving	Above	50 (84.8)	53 (91.4)	51 (89.5)	Reference		Reference		Reference	
	Close to	6 (10.2)	3 (5.2)	6 (10.5)	0.319	0.247	0.332	0.395	0.974	0.944
	Below	3 (5.1)	2 (3.4)	0 (0)	0.634	0.509	0.984	0.954	0.954	0.955
Personal and social	Above	51 (86.4)	45 (77.6)	52 (91.2)	Reference		Reference		Reference	
	Close to	7 (11.9)	10 (17.2)	3 (5.3)	0.345	0.384	0.047	0.077	0.227	0.188
	Below	1 (1.7)	3 (5.2)	2 (3.5)	0.288	0.301	0.541	0.511	0.587	0.735
Number of domain scoring close to the threshold	0	37 (62.7)	40 (69.0)	44 (77.2)	Reference		Reference		Reference	
	1	14 (23.7)	11 (19.0)	5 (8.8)	0.526	0.481	0.119	0.058	0.034	0.522
	≥2	8 (13.6)	7 (12.1)	8 (14.0)	0.742	0.661	0.982	0.741	0.752	0.456
Number of domain scoring below the threshold	0	52 (88.1)	54 (93.1)	54 (94.7)	Reference		Reference		Reference	
	1	3 (5.1)	1 (1.7)	3 (5.3)	0.340	0.338	0.356	0.427	0.964	0.786
	≥2	4 (6.8)	3 (5.2)	0 (0.0)	0.697	0.622	0.954	0.938	0.947	0.947

^1^ Data were described by the frequency (percentage %). sn-2 = the high sn-2 palmitate infant formula, in which 46.3% of the palmitic acid (PA) was esterified to the sn-2 position, BF = breast-fed, and Control = the infant formula, in which 10.3% of the PA was esterified to the sn-2 position. ^2^ A multinomial logistic regression was performed unadjusted. ^3^ A multinomial logistic regression was performed adjusted for the maternal education level.

**Table 4:** Associations of scoring close to and below the typical development threshold for the ASQ-3 domains with the relative abundance of fecal Bifidobacteria.

Developmental Domain	Compared with the Typical Development Threshold	OR (95%CI) ^1^	*p*
Communication	Above	Reference	
	Close to	1.003 (0.961–1.047)	0.888
	Below	0.913 (0.798–1.045)	0.186
Gross motor	Above	Reference	
	Close to	0.989 (0.925–1.057)	0.74
	Below	0.622 (0.287–1.348)	0.229
Fine motor	Above	Reference	
	Close to	0.997 (0.960–1.036)	0.889
	Below	0.839 (0.654–1.077)	0.168
Problem–solving	Above	Reference	
	Close to	0.952 (0.880–1.030)	0.218
	Below	0.978 (0.900–1.062)	0.595
Personal and social	Above	Reference	
	Close to	0.982 (0.938–1.029)	0.452
	Below	1.051 (0.988–1.117)	0.116
Number of domain scoring close to the threshold	0	Reference	
	1	0.947 (0.901–0.996)	0.034
	≥2	1.001 (0.964–1.039)	0.962
Number of domain scoring below the threshold	0	Reference	
	1	1.017 (0.962–1.075)	0.554
	≥2	0.961 (0.884–1.045)	0.352

^1^ Multinomial logistic regression was performed adjusted for treatment and the maternal education level. OR: odds ratio and CI: confidence interval.

## Data Availability

The data presented in this study are available on request from the corresponding author. The data are not publicly available due to restrictions of ethics.

## Supplementary Materials

The following are available online at https://www.mdpi.com/2072-6643/13/2/693/s1: Table S1: Infants’ weights, lengths, or head circumferences. Table S2: ASQ score at week 16 across the feeding groups. Table S3: ASQ score at week 24 across the feeding groups. Table S4: Comparison of the ASQ-3 scores with the typical development threshold at 24 weeks among the feeding groups.
