# Supplementary material for: Neurodevelopmental Outcomes and Gut Bifidobacteria in Term Infants Fed an Infant Formula Containing High sn-2 Palmitate: A Cluster Randomized Clinical Trial
Source: Nutrients. 2021 Feb 22;13(2):693. doi: 10.3390/nu13020693 (PMC7926808; doi:10.3390/nu13020693)
Supplement: Supplementary file 1 [file nutrients-13-00693-s001.zip › Table S2.docx]

Supplementary table 2 ASQ score at week 16 across feeding groups

|  | sn-2 | Control | HM | P | | | |
| --- | --- | --- | --- | --- | --- | --- | --- |
|  |  |  |  |  | **Sn-2 vs Control** | **Sn-2 vs HM** | **Control vs HM** |
| Communication | 50.0 (40.0, 55.0) | 45.0 (40.0, 55.0) | 50.0 (45.0, 55.0) | Unadjusted^b^ | 0.278 | 0.510 | 0.080 |
|  |  |  |  | Adjusted^c^ | 0.287 | 0.442 | 0.122 |
| Gross motor | 55.0 (50.0, 60.0) | 50.0 (45.0, 60.0) | 55.0 (55.0, 60.0) | Unadjusted^b^ | 0.225 | 0.285 | 0.051 |
|  |  |  |  | Adjusted^c^ | 0.232 | 0.221 | 0.061 |
| Fine motor | 50.0 (45.0, 60.0) | 45.0 (35.0, 55.0) | 55.0 (45.0, 60.0) | Unadjusted^b^ | 0.023 | 0.250 | 0.019 |
|  |  |  |  | Adjusted^c^ | 0.021 | 0.096 | 0.062 |
| Problem-solving | 50.0 (45.0, 60.0) | 50.0 (40.0, 60.0) | 55.0 (50.0, 60.0) | Unadjusted^b^ | 0.582 | 0.066 | 0.033 |
|  |  |  |  | Adjusted^c^ | 0.527 | 0.053 | 0.054 |
| Personal and social | 50.0 (40.0, 60.0) | 45.0 (40.0, 60.0) | 55.0 (45.0, 60.0) | Unadjusted^b^ | 0.370 | 0.873 | 0.184 |
|  |  |  |  | Adjusted^c^ | 0.351 | 0.459 | 0.078 |
| Total | 255.0(230.0, 280.0) | 240.0(210.0, 270.0) | 270.0(225.0, 285.0) | Unadjusted^b^ | 0.072 | 0.253 | 0.051 |
|  |  |  |  | Adjusted^c^ | 0.071 | 0.141 | 0.084 |

1. -.Data were described by median (25th, 75th percentiles).
2. Wilcoxon Rank test was performed.
3. Partial correlation analysis was performed with the maternal education level included as the covariate.

sn-2= the high sn-2 palmitate infant formula, in which 46.3% of the PA was esterified to the sn-2 position; HM=human milk;

Control=the infant formula containing a standard vegetable oil mixture, in which 10.3% of the PA was esterified to the sn-2 position.
