# Supplementary material for: Neurodevelopmental Outcomes and Gut Bifidobacteria in Term Infants Fed an Infant Formula Containing High sn-2 Palmitate: A Cluster Randomized Clinical Trial
Source: Nutrients. 2021 Feb 22;13(2):693. doi: 10.3390/nu13020693 (PMC7926808; doi:10.3390/nu13020693)
Supplement: Supplementary file 1 [file nutrients-13-00693-s001.zip › Table S1.docx]

Supplementary Table 1 Infants’ weights, lengths, or head circumferences

|  | sn-2 | Control | HM | P | |
| --- | --- | --- | --- | --- | --- |
|  |  |  |  | Sn-2 vs control | Sn-2 vs HM |
| Baseline | | | | | |
| Weight(kg) | 3.3(3.1, 3.6) | 3.4(3.1, 3.6) | 3.4(3.1, 3.6) | 0.793 | 0.158 |
| Height (cm) | 50.0(50.0,51.0) | 50.0(50.0, 51.0) | 50.0(50.0, 51.0) | 0.830 | 0.700 |
| Week 16 | | | | | |
| Weight(kg) | 7.3(6.8, 8.0) | 7.2(6.5, 8.0) | 7.0(6.5, 7.7) | 0.722 | 0.196 |
| Height (cm) | 64.0(62.0, 66.0) | 63.0(61.0, 65.0) | 64.0(61.5, 66.0) | 0.368 | 0.825 |
| Week 24 | | | | | |
| Weight(kg) | 8.5(7.8, 9.0) | 8.0(7.5, 9.0) | 8.0(7.5, 8.8) | 0.655 | 0.661 |
| Height (cm) | 68.0(66.0, 70.0) | 67.0(65.0, 70.0) | 67.5(66.0, 70.0) | 0.454 | 0.202 |
