# Supplementary material for: Neurodevelopmental Outcomes and Gut Bifidobacteria in Term Infants Fed an Infant Formula Containing High sn-2 Palmitate: A Cluster Randomized Clinical Trial
Source: Nutrients. 2021 Feb 22;13(2):693. doi: 10.3390/nu13020693 (PMC7926808; doi:10.3390/nu13020693)
Supplement: Supplementary file 1 [file nutrients-13-00693-s001.zip › Table S4.docx]

Supplementary Table 4 Comparison of ASQ-3 scores with the typical development threshold at 24 weeks among feeding groups

|  | Compared with the typical development threshold | Control | sn-2 | HM | P | | | | | |
| --- | --- | --- | --- | --- | --- | --- | --- | --- | --- | --- |
|  |  |  |  |  | **sn-2 vs Control** | | **sn-2 vs HM** | | **Control vs HM** | |
|  |  |  |  |  | Unadjusted ^b^ | Adjusted ^c^ | Unadjusted ^b^ | Adjusted ^c^ | Unadjusted ^b^ | Adjusted ^c^ |
| Communication | Above | 54(91.5) | 53(91.4) | 57(100.0) | Reference | | Reference | | Reference |  |
|  | Close to | 4(6.8) | 4(6.9) | 0(0.0) | 0.980 | 0.886 | 0.921 | 0.948 | 0.922 | 0.911 |
|  | Below | 1(1.7) | 1(1.7) | 0(0.0) | 0.990 | 0.931 | 0.961 | 0.938 | 0.961 | 0.957 |
| Gross motor | Above | 49(83.1) | 54(93.1) | 51(89.5) | Reference | | Reference | | Reference |  |
|  | Close to | 7(11.9) | 3(5.2) | 5(8.8) | 0.188 | 0.173 | 0.391 | 0.642 | 0.635 | 0.659 |
|  | Below | 3(5.1) | 1(1.7) | 1(1.8) | 0.307 | 0.260 | 0.923 | 0.545 | 0.368 | 0.515 |
| Fine motor | Above | 55(93.2) | 55(94.8) | 57(100.0) | Reference | | Reference | | Reference |  |
|  | Close to | 4(6.8) | 1(1.7) | 0(0.0) | 0.065 | 0.065 | 0.961 | 0.957 | 0.941 | 0.807 |
|  | Below | 0(0.0) | 2(3.5) | 0(0.0) | 0.993 | 0.959 | 0.944 | 0.940 | — | 0.954 |
| Problem-solving | Above | 53(89.8) | 51(87.9) | 54(94.7) | Reference | | Reference | | Reference |  |
|  | Close to | 4(6.8) | 6(10.3) | 2(3.5) | 0.511 | 0.478 | 0.199 | 0.093 | 0.475 | 0.516 |
|  | Below | 2(3.4) | 1(1.7) | 1(1.8) | 0.598 | 0.529 | 0.989 | 0.582 | 0.609 | 0.727 |
| Personal and social | Above | 51(86.4) | 50(86.2) | 52(91.2) | Reference | | Reference | | Reference |  |
|  | Close to | 7(11.9) | 6(10.3) | 5(8.8) | 0.820 | 0.862 | 0.825 | 0.556 | 0.656 | 0.676 |
|  | Below | 1(1.7) | 2(3.5) | 0(0.0) | 0.566 | 0.619 | 0.944 | 0.960 | 0.961 | 0.951 |
|  |  |  |  |  |  |  |  |  |  |  |
| Number of domain scoring close to the threshold | 0 | 42(71.2) | 46(79.3) | 48(84.2) | Reference | | Reference | | Reference |  |
|  | 1 | 9(15.3) | 8(13.8) | 7(12.3) | 0.694 | 0.725 | 0.451 | 0.396 | 0.404 | 0.404 |
|  | ≥2 | 8(13.6) | 4(6.9) | 2(3.5) | 0.227 | 0.237 | 0.451 | 0.261 | 0.076 | 0.090 |
| Number of domain scoring below the threshold | 0 | 55(93.2) | 52(89.7) | 56(98.2) | Reference | | Reference | |  |  |
|  | 1 | 2(3.4) | 5(8.6) | 0(0.0) | 0.258 | 0.297 | 0.942 | 0.937 | 0.944 | 0.960 |
|  | ≥2 | 2(3.4) | 1(1.7) | 1(1.8) |  |  | 1.000 |  | 0.607 | 0.727 |

1. Data were described by frequency (percentage %).
2. Multinomial logistic regression was performed unadjusted.
3. Multinomial logistic regression was performed adjusted for maternal education level.

sn-2= the high sn-2 palmitate infant formula, in which 46.3% of the PA was esterified to the sn-2 position; HM=human milk; Control=the infant formula containing a standard vegetable oil mixture, in which 10.3% of the PA was esterified to the sn-2 position.
