# Supplementary material for: Neurodevelopmental Outcomes and Gut Bifidobacteria in Term Infants Fed an Infant Formula Containing High sn-2 Palmitate: A Cluster Randomized Clinical Trial
Source: Nutrients. 2021 Feb 22;13(2):693. doi: 10.3390/nu13020693 (PMC7926808; doi:10.3390/nu13020693)
Supplement: Supplementary file 1 [file nutrients-13-00693-s001.zip › Table S3.docx]

Supplementary table 3 ASQ score at week 24 across feeding groups

|  | sn-2 | Control | HM | P | | | |
| --- | --- | --- | --- | --- | --- | --- | --- |
|  |  |  |  |  | **Sn-2 vs control** | **Sn-2 vs HM** | **Control vs HM** |
| Communication | 50.0 (45.0, 60.0) | 55.0(45.0, 60.0) | 55.0(50.0, 60.0) | Unadjusted^b^ | 0.903 | 0.248 | 0.082 |
|  |  |  |  | Adjusted^c^ | 0.902 | 0.115 | 0.203 |
| Gross motor | 45.0 (40.0, 50.0) | 40.0(30.0, 50.0) | 50.0(35.0, 55.0) | Unadjusted^b^ | 0.070 | 0.241 | 0.056 |
|  |  |  |  | Adjusted^c^ | 0.074 | 0.135 | 0.053 |
| Fine motor | 55.0 (45.0, 60.0) | 50.0(45.0, 60.0) | 55.0(50.0, 60.0) | Unadjusted^b^ | 0.279 | 0.522 | 0.063 |
|  |  |  |  | Adjusted^c^ | 0.282 | 0.774 | 0.299 |
| Problem-solving | 55.0 (40.0, 60.0) | 50.0(45.0, 60.0) | 55.0(50.0, 60.0) | Unadjusted^b^ | 0.482 | 0.804 | 0.212 |
|  |  |  |  | Adjusted^c^ | 0.542 | 0.795 | 0.145 |
| Personal and social | 42.5(35.0, 50.0) | 45.0(35.0, 50.0) | 50.0(45.0, 55.0) | Unadjusted^b^ | 0.346 | 0.064 | 0.072 |
|  |  |  |  | Adjusted^c^ | 0.367 | 0.134 | 0.157 |
| Total | 245.0(215.0, 270.0) | 240.0(215.0, 260.0) | 255.0(235.0, 275.0) | Unadjusted^b^ | 0.203 | 0.097 | 0.051 |
|  |  |  |  | Adjusted^c^ | 0.367 | 0.115 | 0.093 |

1. Data were described by median (25th, 75th percentiles).
2. Wilcoxon Rank test was performed.
3. Partial correlation analysis was performed with the maternal education level included as the covariate.

sn-2= the high sn-2 palmitate infant formula, in which 46.3% of the PA was esterified to the sn-2 position; HM=human milk;

Control=the infant formula containing a standard vegetable oil mixture, in which 10.3% of the PA was esterified to the sn-2 position.
